# Leuprolide-Induced Hyperosmolar Hyperglycemic State in an Elderly Patient: A Case Report and Literature Review

**DOI:** 10.7759/cureus.26993

**Published:** 2022-07-18

**Authors:** Eltaib Saad, Akram Babkir, Abdalaziz M Awadelkarim, Faisal Qureshi

**Affiliations:** 1 Internal Medicine, AMITA Saint Francis Hospital, Evanston, USA; 2 Internal Medicine, Wayne State University Detroit Medical Center, Detroit, USA; 3 Internal Medicine and Endocrinology, AMITA Health Saint Joseph Hospital, Chicago, USA

**Keywords:** gonadotropin-releasing hormone agonists, androgen-deprivation therapy, leuprolide, impaired glycemic control, hyperosmolar hyperglycemic state

## Abstract

We present a novel case of severe hyperosmolar hyperglycemic derangement in an elderly patient - without a known history of diabetes mellitus - after the first injection of leuprolide for the treatment of metastatic prostate adenocarcinoma. Whilst the available literature provided accumulative evidence of an association between insulin resistance and the use of gonadotropin-releasing hormone (GnRH) agonists, the initial presentation of leuprolide-induced impaired glycemic tolerance with a hyperosmolar hyperglycemic state (HHS) represents a clinical rarity that was seldom reported. A literature review was conducted to explore the underlying mechanisms of leuprolide-associated glucose intolerance. Screening for diabetes is recommended for patients receiving leuprolide therapy to identify at-risk patients and close glycemic monitoring is warranted in diabetic patients to minimize serious complications from poor glycemic control induced by leuprolide.

## Introduction

The use of gonadotropin-releasing hormone (GnRH) agonists in metastatic prostate cancer has increased in the last two decades [1]. The systemic effects of GnRH resulting from a low testosterone state (such as hot flashes, gynecomastia, osteoporosis, and hypogonadism) are well-known [1, 2]. Furthermore, the recent literature has explored the potential effects of GnRH agonists on glucose homeostasis and insulin resistance [1-3]. In this report, we present an interesting case of hyperosmolar hyperglycemic state (HHS) as a presenting feature of GnRH-induced impaired glycemic control in an elderly patient with no previous history of diabetes mellitus within a short time interval following the first injection of leuprolide for the treatment of metastatic prostate cancer. A brief review of the literature that explored the presumed association between androgen-deprivation therapy (ADT) and impaired glucose tolerance was included in this report, with a summary of postulated theories explaining the mechanisms of acquiring insulin resistance that was induced by ADT.

## Case presentation

A 75-year-old **African American **male patient was brought to our emergency department with generalized weakness, nausea, and anorexia for four days associated with an altered mental status for one day as per the patient’s son. Past medical history was significant for essential hypertension, gout, and prostate adenocarcinoma status post-prostatectomy and radiotherapy in 2016 with a recent metastatic recurrence to the liver, which was diagnosed three months prior to his index presentation. Regular medications included amlodipine 10 mg daily, allopurinol 100 mg daily, and bicalutamide 50 mg daily. The patient received the first injection of leuprolide two months prior to his present admission. He was scheduled for a three-monthly leuprolide 22.5 mg depot intramuscular therapy. There was no history of chest pain, shortness of breath, or cough. The rest of the systemic review was non-contributory. There was neither known personal nor family history of diabetes mellitus.

The initial evaluation revealed an ill-looking afebrile elderly patient with a pulse rate of 115 beats per minute and blood pressure of 77/55 mmHg. The patient was confused, drowsy, and difficult to arouse. The patient’s body mass index (BMI) was 19.9 kg/m². Physical examination revealed dry mucous membranes with generalized abdominal tenderness on deep palpation without focal guarding. Neurological examination demonstrated no focal weakness. No acanthosis was identified on the examination. The rest of the systemic examination was unremarkable. Initial laboratory results showed a blood glucose of 996 mg/dL (reference range 90-110 mg/dL) with normal serum ketones level, normal serum bicarbonate (22.0 mmol/L, reference 22.0-26.0 mmol/L), and a normal anion gap. Serum osmolality was 330 mOsm/Kg (reference limits 270-300 mOsm/Kg). Table 1 summarizes the initial laboratory results for the reported patient on arrival.

**Table 1:** Pertinent laboratory values on admission

Blood test	Patient’s result	Reference result
Serum glucose (Point-of-Care [POC])	996 mg/dL	70-110 mg/dL
Serum ketones	0.6 mmol/L	< 0.6 mmol/L
Venous lactate	2.1 mmol/L	0.6- 2.1 mmol/L
Venous pH	7.30	7.31- 7.41
Serum bicarbonate	24.0 mmol/L	21.0 -28.0 mmol/L
Serum anion gap	10.0 mmol/L	6.0- 14.0 mmol/L
Serum osmolality	330 mOsm/Kg	280-300 mOsm/Kg
Serum creatinine	2.30 mg/dL	0.6- 1.3 mg/dL
Serum sodium	138 mmol/L	133- 144 mmol/L
Serum chloride	99 mmol/L	98- 107 mmol/L
Serum potassium	4.6 mmol/L	3.5- 5.2 mmol/L
Serum calcium	9.6 mg/dL	8.6- 10.3 mg/dL
Serum magnesium	1.4 mg/dL	1.5- 2.6 mg/dL
Serum phosphate	4.1 mg/dL	2.5- 4.5 mg/dL
Serum uric acid	6.9 mg/dL	4.4- 7.6 mg/dL
Hemoglobin	17.5 g/dL	13.0-17.0 g/dL
Hemoglobin A1c	13.5%	< 5.7%
White cells count (WCC)	12.5 k/mm cu	4.0- 11.0 k/mm cu
Serum albumin	3.8 g/dL	3.5- 5.7 g/dL
Procalcitonin	0.41 ng/ mL	0.20- 0.49 ng/mL

Hemoglobin A1c was 13.5% (reference < 5.7%). Computed tomography of the abdomen revealed no acute intra-abdominal process apart from the metastatic hepatic nodules. Notably, the patient had a recent admission to our facility three months prior to index presentation, when the recurrent prostate cancer was diagnosed, and serial fasting glucose readings ranged between 90-100 mg/dL. There were no previous hemoglobin A1c results in our institution or the available outside records.

A diagnosis of hyperosmolar hyperglycemic state (HHS) was made. The patient was admitted to the intensive care unit for aggressive fluids resuscitation, continuous insulin infusion, and optimization of electrolytes disturbances as per our institution-based protocol. The patient’s glucose levels normalized after 12 hours of insulin infusion, and mentation improved remarkably. The following day, the patient was alert, returned to baseline mental status per family, and resumed oral feeding. Endocrinology consultation recommended fast-acting insulin (lispro) six units three times with meals and basal insulin (levemir) 12 units twice daily alongside intensive diabetic education.

## Discussion

Leuprolide is a gonadotropin-releasing hormone (GnRH) agonist that has been authorized to be used in a range of benign and neoplastic conditions (i.e., challenging endometriosis, central precocious puberty, and advanced prostate cancer) [1-3]. The recent employment of androgen-deprivation therapy (ADT) with GnRH agonists, either as monotherapy or in combination with an anti-androgen (AA) agent, has significantly impacted the outcome of metastatic prostate adenocarcinoma with prolonged disease-free intervals and improved the quality of life [1].

In fact, in the year 2010, the Food and Drug Administration (FDA) warned patients with prostate cancer treatment who are using GnRH agonists about an increased risk of diabetes while they are receiving these medications [4]. Nevertheless, the effects of GnRH agonists on glucose metabolism were not yet fully understood until the recent literature elucidated these effects [3, 5, 6]. A meta-analysis of several cohorts published in 2015 revealed a 36% increased risk of developing diabetes following ADT for men with prostate cancer (a pooled relative risk (RR) of 1.36, 95% CI: 1.17-1.58) [3].

A large nation-based Canadian study was conducted in a cohort of men aged above 66 years who received at least 6 months of ADT and reported an increased risk of type 2 diabetes mellitus (T2DM) (RR 1.16, 95% CI: 1.11- 1.21), but the latter cohort did not assess the effects of GnRH and AA separately as it combined all modalities of ADT as a single exposure variable [5]. Interestingly, men treated with GnRH agonists are not only more likely to have locally advanced or metastatic disease, but they are also prone to have more comorbidities than those treated with AA, hence, patients on GnRH agonists may also be at higher risk of T2DM than those on AA [6].

Early observational studies have shown more tendency of hypogonadotropic males to develop diabetes mellitus than their eugonadotropic counterparts [7]. The available evidence remains limited regarding the association between ADT and insulin resistance [1, 3, 6]. Multiple theories were postulated to explain this presumed association [1, 3, 6, 8]. It was suggested that ADT-induced increase in fat mass adversely affects peripheral insulin sensitivity [3, 6]. Furthermore, it was theorized that testosterone might directly stimulate insulin gene expression in beta-pancreatic cells, and thus ADT may reduce de-novo synthesis and release of insulin [8]. Additionally, testosterone has been assumed to regulate cell lineage determination by inhibiting the adipogenic lineage, thus providing insight into increased fat deposition and visceral obesity associated with ADT [3]. 

An early Japanese cohort conducted by Suzuki K et al [1] enrolled 52 patients with prostate cancer (13 of them were diabetics) who received a combination of GnRH and an AA for at least three months [1]. A statistically significant elevation in mean blood glucose levels was observed after receiving ADT, with less than one-third of patients (28%) experiencing more than a 30 mg/dl increase in their mean blood glucose levels after receiving ADT [7]. Of note, greater changes were more pronounced in patients with concurrent diabetes and those with higher BMI [1]. Suzuki K et al recommended rigorous glycemic monitoring for those receiving ADT, particularly if they were diabetic or obese [1]. Another American study evaluated potential long-term glucose tolerance among a cohort of eighteen patients with recurrent or metastatic prostate cancer who received ADT for at least one year and compared their metabolic profile (fasting glucose levels, fasting insulin levels, and homeostatic model of insulin resistance (HOMA-IR)) with two age-matched groups (non-ADT-receiving prostate cancer group and a normal control group) [7]. After 12 months of ADT therapy, the mean levels of fasting blood glucose and fasting serum insulin levels in HOMA-IR were found to be significantly higher in the ADT-receiving group than in the other two groups [7], and the cohort concluded a risk of insulin resistance and hyperglycemia as a long-term effect of ADT, which was largely independent of age and BMI. The worse cardiovascular mortality observed in males with hypogonadism was partly attributed to increased insulin resistance associated with low testosterone levels [8].

The authors postulated leuprolide therapy as the culprit of poor glycemic control and onset of HHS in this reported patient given a robust temporal association between a recent leuprolide exposure and the new onset of severe hyperglycemia. Although our patient was not known to be diabetic and had normal fasting glucose levels prior to leuprolide introduction, the possibility of undiagnosed diabetes that might have been progressively worsened by leuprolide use cannot be entirely ruled out. Nevertheless, this case is considered the first - to the best of the authors' knowledge - to report HHS as a presenting feature of leuprolide-induced impaired glycemic control in a patient without a previous history of diabetes within a short-term interval following the first injection of leuprolide. Diabetes screening with close glycemic monitoring is recommended for patients receiving leuprolide to minimize the occurrence of serious diabetic complications such as the HSS described in this patient [4].

## Conclusions

We described a novel case of hyperosmolar hyperglycemic state (HHS) in an elderly patient with no previous history of diabetes mellitus that was induced by leuprolide therapy, in keeping with the current literature that reported an association between the use of GnRH agonists and the development of diabetes. Screening for diabetes is recommended for patients receiving GnRH agonists to minimize the risk of complications like HSS reported in this presented case.
